# Supplementary material for: Patterns of Intron Gain and Loss in Fungi
Source: PLoS Biol. 2004 Nov 30;2(12):e422. doi: 10.1371/journal.pbio.0020422 (PMC532390; doi:10.1371/journal.pbio.0020422)
Supplement: Table S1 — Also available at http://genes.mit.edu/NielsenEtAl/. (4.3 MB ZIP). [file pbio.0020422.st001.zip › NielsenEtAl/html/74.html]

AN3665.1.NCU02632.1.MG04433.1.FG01006.1


```
 CLUSTAL W (1.82) Multiple Sequence Alignments - Introns Inserted


Sequence 1: NCU02632.1	1118 aa
Sequence 2: FG01006.1	1079 aa
Sequence 3: MG04433.1	1103 aa
Sequence 4: AN3665.1	1201 aa
Alignment Length: 1210 aa
Number Identitical Residues: 374 aa
Alignment Score (without introns) 19921


MG04433.1 	----M~SSPSR--FSWRRANIDPAQANDISTTSNNNDSPTAGLISPP-----NGPGETRD
NCU02632.1	----M~SSPAAGFSSWRRSPRRSSSVSQYASAQEEIESSSPSKTIPTRPCQDPTSNETHQ
FG01006.1 	----M~SSPLN-FTPWRR--RALSTASQRDNEQAQLPREEPSTSPPQ---HGHSPNLPIA
AN3665.1  	MQHEQ0PAKAQTHQSLGPPILFCQTLSLALPTTAQLFHLTTLISLPSHVVVCTSPALRIA
          	 . .  .:      .   .       .       :     .    *        .     

MG04433.1 	R-SLSFGSAS------YREPIRSFIHGSMRDRYT1P--------------VDSAQHARSV
NCU02632.1	LYSTSVNSVA------HREPLRSFIHGSVRENLA1P--------------LDSYQYAQSV
FG01006.1 	RPSSTSSAAG------HREPIRSFIHGSMRDSLA1P--------------IDSIQNARSV
AN3665.1  	SIAISLASSPQLPLEWIRNPTTTAIEAWIANQHP~SPLLFVPRQLWAFWGAGSAQTRKPP
          	  : :  :  . . .  *:*  : *.. : :  . ..    . .  :  . .* *  :. 

MG04433.1 	REDTAELASYLLSDQNNRRPPSFLNRPRSSSSRFSRDAPQVFSPAAV~EDEALSP----A
NCU02632.1	RQDTAELANYFLSDPKDRQSPAFLDRRRSSISQQTVLGHIAQATGTE~DAEEGDG----R
FG01006.1 	RQDTAELATYLLSDPKTQQNSSVLQRARVSYQDS------IDSEAVD~DSSIAEN----G
AN3665.1  	TLHQYQMTMLLPTQSIPSLGLVALLAFRSRNPKYLEGQGRVTGHRAD1PAHYSSHGLREQ
          	  .  :::  : ::         *   *              .  .       . .  . 

MG04433.1 	KDTEIIEEISEPPSPEPVEEGEDFNGAGPSILANMLRRSPPEDNLKDLDRQPPGDLHIGA
NCU02632.1	HQDALNVNISEPLSP---ETDVESTAEGPSMLTTMLKRSPPSTIYMPASRSDEGYEEDNS
FG01006.1 	RSSKTILEVPEPPSPDGNEDDLDADA-GPSVLANLLKRSPPQSIAP--DRSNEQHGDDHV
AN3665.1  	TAELASLALTREPRALPQAMEIFRPVDGSAESVAPLSLSGPDRTQEMGHTPTVHSGGSSV
          	        :..   .            *.:  .  *  * *.                  

MG04433.1 	MSPG-LRDYGSLHKKQDLHEQERENNMSETTPLLGVRPRQSIR--------LNGTSLTSD
NCU02632.1	WENGEGSRRGSWKHPELVQPTEREAEAADTSDEHAPLLRTTSREAQHDYGMVNGSGHDAD
FG01006.1 	EDN------------QAEQDPRRTSHAEDATEQTPLLARMTS----------GGRRSYSD
AN3665.1  	LTALIRDPSSSVSREGQERTGDRNEEGERAHAVGPSAPGNADEEEDTSTEWTSLLAKTPS
          	         .:       :   *  .   :          :  . . .    .     ..

MG04433.1 	LESQKVADP----PKSGFLHGLAGVRHSLESHVSAFFRVAREPKCWDKRAIWQAAVVDPV
NCU02632.1	IEDQKS-------PKRRWTTRLAASARDRWDHIASITKVAATPKRWDRHALWKNVIVTPV
FG01006.1 	LEGQKAQ------SKPSWFSGFVEVGHRMEERIS----VAVNPKKWDRQALWNNVVLTPT
AN3665.1  	RPSRNYGGAGDVESQVYAPKETQRTLPSALTTVSTCFQVLAHPKSWDRRTVWRQGVVRPF
          	  .::  .... ..:                 :::   *   ** **::::*.  :: * 

MG04433.1 	ACLPAVIVGLLLNILDALSYG1MILFPLGNPIFSNLGPAGISIFYVSTIVSQLTYSMG-S
NCU02632.1	KCLPAVIVGLLLNILDALSYG~MILFPLANPIFSSLGSAGISIFYISTIISQLTFSLG-S
FG01006.1 	SCLPAVSVGLLLNILDALSYG1MILFPLGKPIFSHLGSAGISVFYVSTIVSQITFSSG-S
AN3665.1  	SLLPSVFLGLLLNILDALSYG~MILFPLGEPIFSHLGSDGISMFYVSTIIAQLVFSCGGS
          	  **:* :************* ******.:**** **. ***:**:***::*:.:* *.*

MG04433.1 	IFKGSVGSEM0IEVVPFFHSMASTITTSIGQDDPDAVIATTIVAYAVSSMMTGIVFFLMG
NCU02632.1	VFKGGVGSEL0IEVVPFFHSMAGTITEIVGEDDPEAVIATTITSYALSSMLTGTVFYLMG
FG01006.1 	IFKGAVGSEL0IEVVPFFHNMAGKITEVVGEENPDAVIATTIVSFSVSAMMTGLVFYLMG
AN3665.1  	IFRGGIGSEM~IEVVPFFHQMAFTILNRVGQDNPKSVIATTILAFSVSSVLTGLVFFLMG
          	:*:*.:***: ********.** .*   :*:::*.:****** ::::*:::** **:***

MG04433.1 	YFKFGYIVGFIPRHILIGCIGGVGWFLVATGFEVSARIEGSLEYDLATLNKLINPETIPL
NCU02632.1	KFKFGYLVGFIPRHILIGCIGGVGWFLIATGFEVTARMDGSLNYDLDTLKRLIQPDTIPL
FG01006.1 	RFKVGYMVGFIPRHILIGCIGGVGWFLIATGFEVSARLDGSLQYNLDTLKQLSDPATVPL
AN3665.1  	TCKIGSLIGFFPRHILIGCIGGVGFFLILTGLEVSARLPGSFEFDIPTMQKLFNLAALAL
          	  *.* ::**:*************:**: **:**:**: **::::: *:::* :  ::.*

MG04433.1 	WATPLALAVALVYLQKKIKSTYFLPCFILMIPIVFYIFVFSLDSLNPESLRESGWIFEGA
NCU02632.1	WTVPLGLAIILFYGQTKITSKYFLPLYILTIPALFYLVLLILGGPDQDTLQKHGWVFEGP
FG01006.1 	WVVPLVLAIILFYGQSKITSKFFLPLFILAIPLIFYFFVLALDTLHIDTLRDHGWIFEGP
AN3665.1  	WVTPLLLAIGLLVLKRFVRSNYLVGAYFIVVALCFYIVKFIAHIP-MDSLRNSGWVFDAP
          	*..** **: *.  :  : *.:::  ::: :.  **:. :       ::*:. **:*:..

MG04433.1 	ASDEPWWYFYTLYK1FHLVRWDVLAETIPAMLALTFFGILHVPINVPALAMSAGEDNVDL
NCU02632.1	PPGEPWWYFYTLYK1FQKVDWSAIIQCIPAMFALTFFGILHVPINVPALAQNIGEDHADL
FG01006.1 	PSGEPWWYFYTLYK1FHLVRWDAVIECVPAMLALTFFGILHVPINVPALALNCGEDHADL
AN3665.1  	SSSNPWYHFYTLYD1FSAVHWPAFVDTIPAMFALTFFGILHVPINVPALGISTGEDNLNV
          	...:**::*****. *  * * .. : :***:*****************. . ***: ::

MG04433.1 	NHELKLHGYSNFLSGLTGSIQ~NYLVYANTVMFMRSGANSRLAGFELAALTFGVLLVGPR
NCU02632.1	DHELKLHGYSNFLSGCFGSIQ~NYLVYANSVFFMRSGGNTRLAGIMLAVLTFLVMMIGPS
FG01006.1 	DKELRLHGYSNFLSGCFGSIQ~NYLVYANTVFFMRSGGNSRLAGYMLAAATFGIMVIGPS
AN3665.1  	DRELIAHGVTNALSGFAGSIQ0NYLVYTNSLLFIDSGGNSRLAGVMLAGATAGIMLVGPV
          	::**  ** :* ***  **** *****:*:::*: **.*:****  **  *  ::::** 

MG04433.1 	LIGIIPVMMVGCLIFVLGFELLSEALWHPRKKLKTLEYLT~VVAIILIMGIYDFVIGIGC
NCU02632.1	LIGFIPVMMVGVLIFDLGFELLIEAVWQPRKKLKWLEYMT~VLVIVVVMGTYDFVIGIGV
FG01006.1 	LIGYIPVMMVGTLIYDLGFELLLEALWLPRKKLKLAEYLT~VVVIVLVMGIHDFVVGIGV
AN3665.1  	IVGFIPVMVVGALIFLLGIELMEEALVDTWGKLHRHEYLT0VVIIVATMGVWDFVAGILV
          	::* ****:** **: **:**: **:  .  **:  **:* *: *:  **  *** **  

MG04433.1 	GILIAFVTLVFQTSQVSAVRAGWSGDVVGSTVRRNPSQQNYLKQVGSQIHVVKLSGYLFF
NCU02632.1	GILCAFAALIIQTSRISAVRAIYSGEVVGSTVRRNPTQQHYLRDAGKQVNIIKLAGYLFF
FG01006.1 	GILLAFVSLILQTSRVSAIRGNYSGDIVTSTVRRNPSQQRYLHEVGRQIYIAKLTGYLFF
AN3665.1  	GIILACLSFVVQTSRKSAIRATYSGKVAGSTVRRPPIQQRYLKEAGQQTLILKLGGYLFF
          	**: *  :::.***: **:*. :**.:. ***** * **.**::.* *  : ** *****

MG04433.1 	GTIVGVEEKIREIIADETFRQRPIRFLVVDLHHVAGCDYSAGEAFGTLSRLLKKKNIEFI
NCU02632.1	GTIVSVEEKIRAMISDEEFNHRPIRFLIIDLWLVNGVDYSAGEVFNTISRLLNHKGIELI
FG01006.1 	GTIVSVEERIRALLEDSAFTAQPIKFLILDMWHVTGLDYSAGEAFNTISRLLDNKDVVLV
AN3665.1  	GTIVDVENTMRGLIEDEAFSRLPIRFIILDLCRVYGVDFSAAEAFTRINRILKKRNVRMM
          	****.**: :* :: *. *   **:*:::*:  * * *:**.*.*  :.*:*.::.: ::

MG04433.1 	LSGVDDDGALSHSLRTVGIGEE--VATVLPDLNSALESCENELLKTLYARQE--DASRNP
NCU02632.1	ISGVDTEQELGRHLRAAGLGEDGIDVKLLPELNSALEYCENELLKTLYASQEAGRLPRTA
FG01006.1 	LSGVDSETQLGRNLRAVGLGNDGIEVMMLPNLNSALESCENELLKTLYARQKEINSLRRI
AN3665.1  	ISGLDVGGDVGKSLQNVGLFEPELTVRIFEDLNSALEYCENEYLNVFYSHREA--LLRRK
          	:**:*    :.: *: .*: :    . :: :****** **** *:.:*: ::     *  

MG04433.1 	PTPASANLDVPGQQHTDSASQGPLDMLASSPRRSHLWEAAMQAVNAGLTRQ---NKWAAF
NCU02632.1	ASVPSSSLQVPS---TQSSIQPQFDSLGSSPRRSHLQVAARHALNQQAEQQRGSTRWQSF
FG01006.1 	SAQSAQNLDVPS---NKSSGFSSFDPPFNSPRRNHLAEAARDTLSSIDVQN--PKKWQSF
AN3665.1  	AAP--QNLEVPAIQHRSQSAEG----FVGSPRHQYLQRAATTTLSEDESAILPPAAWSAM
          	.:    .*:**. .  ..:         .***:.:*  **  ::.        .  * ::

MG04433.1 	GEPLRLLLQIFSDLSDKNEDFWFRAVPFFAAKRYAAGDVLYRQGEPAEGFYLLESGIVRM
NCU02632.1	KEPLRLMLQIFQDVSNKNEDFWFRAVGYFKRLQYPAGTVIFRRGEQAEGFYLLEQGMLRA
FG01006.1 	KEPLRLMLQVFQGLSDKNEDFWFPATSYFSRREIPAGTVLFRRGEQANGFYLVERGIMRA
AN3665.1  	RQPLPLLLQTFQGLTSRNEDFWFAACPYFVRTTYAMGTTLFREGDVPNAFYLLESGMLRA
          	 :** *:** *..::.:****** *  :*     . * .::*.*: .:.***:* *::* 

MG04433.1 	EYDLPQGRLCESIVAGTTCGELPFFSDTCRTATAVVERDAVVWLIDREAWAKLQRKEPDV
NCU02632.1	EYDLPQGRLCESIVAGTTCGELPFFSETTRTATCVVERDCVLWMMDREGWEKLQKEQPDV
FG01006.1 	EYDLPQGWLCESIVAGTTLGELPFFSETDRTATAVVERDCVIWLMDRDQWARIQKKEPEI
AN3665.1  	EYDLPQGRYFELIVAGRPCGELPFFSETRRTATVKAEQDCVTWSLDAENWKALKEEEPDI
          	*******   * **** . *******:* ****  .*:*.* * :* : *  ::.::*::

MG04433.1 	AQELLRMGLKITSERMNAMTS~-----------~------HV~LTLAY------------
NCU02632.1	AMELLRIGLKLTSERMSVFTS~-----------~------YT~LTMAG------------
FG01006.1 	GRELLKISLKLTSERMSAITS~-----------~------YI~LTTAG------------
AN3665.1  	ARELLTVSLKLTTERMDSITS2RCCWWVACLGA0ALLGRRFQ0FAAAPTMISRNMQSSLQ
          	. *** :.**:*:***. :**  ..   :. .: :  .  .  :: * :  : . .:: .

MG04433.1 	---------------------
NCU02632.1	---------------------
FG01006.1 	---------------------
AN3665.1  	QIFDVSKHLHSEVDAQMSCHL
          	.  . :.   :. .:. :.
```
